# Rates of out-of-home care among children in Canada: an analysis of national administrative child welfare data

**DOI:** 10.24095/hpcdp.44.4.02

**Published:** 2024-04

**Authors:** Nathaniel J. Pollock, Alexandra M. Oudraogo, Nico Trocm, Wendy Hovdestad, Amy Miskie, Lindsay Crompton, Aime Campeau, Masako Tanaka, Cindy Zhang, Claudie Laprise, Lil Tonmyr

**Affiliations:** 1 Family Violence Epidemiology Section, Health Promotion and Chronic Disease Prevention Branch, Public Health Agency of Canada, Ottawa, Ontario, Canada; 2 School of Arctic and Subarctic Studies, Labrador Campus, Memorial University, Happy Valley-Goose Bay, Newfoundland and Labrador, Canada; 3 School of Social Work, McGill University, Montral, Quebec, Canada; 4 Ministry of Children and Family Services, Government of Alberta, Edmonton, Alberta, Canada; 5 Dalla Lana School of Public Health, University of Toronto, Toronto, Ontario, Canada; 6 cole de sant publique, Dpartement de mdecine sociale et prventive, Universit de Montral, Montral, Quebec, Canada; 7 Faculty of Dental Medicine and Oral Health Sciences, McGill University, Montral, Quebec, Canada

**Keywords:** alternative care, child protective services, epidemiology, foster care, pediatrics, public health surveillance, secondary data, social work

## Abstract

**Introduction:**

As a part of the public health approach to child welfare, data about children placed in out-of-home care are needed to assess population trends, understand drivers of social and health inequities, and examine outcomes for children and families. We analyzed administrative data from Canada to describe the population of children in out-of-home care, and estimate and compare rates of out-of-home care by province/territory, year, sex/gender, age group and placement type.

**Methods:**

We conducted a cross-sectional analysis of point-in-time data from all provinces and territories for the period 2013/2014 to 2021/2022. We used frequencies and percentages to describe the population of children (and youth up to age 21 years) in out-of-home care and estimated overall and stratified rates and rate ratios.

**Results:**

An estimated 61104 children in Canada were in out-of-home care on 31 March 2022. The national rate of out-of-home care was 8.24 children per 1000 population. Rate variations by province/territory were substantial and changed over time. Rates were highest among males and children aged 1 to 3 and 16 to 17 years. Foster homes were the most common type of placement, although kinship homes accounted for an increasing share.

**Conclusion:**

This analysis demonstrated that administrative data can be used to generate national indicators about children involved in the child welfare system. These data can be used for tracking progress towards health and social equity for children and youth in Canada.

**Highlights**

- About 61 104 children were in outof- home care in Canada in 2021/2022; the national rate was 8.24 per 1000.
- Most of the children in out-of-home care (84.3%) were placed in a family-based care setting such as a foster home or with extended family (e.g. in a kinship home).
- The rate of out-of-home care varied by province/territory from 2.72 to 29.60 per 1000 children.
- National administrative child welfare data can be used for public health monitoring.

## Introduction

Children and youth have the right to be healthy, to receive a high standard of care and to be protected from violence and neglect.^1^ Under the United Nations *Convention on the Rights of the Child*, governments have legislated authority to enact these rights.^1^ Using the law to protect children is a responsibility of child welfare systems in many countries.^2^,^3^ In Canada, child welfare legislation and policies are primarily determined by provincial and territorial governments, and services are most often delivered by government departments or ministries and government-funded agencies.^2^,^3^ In 2019, a federal act affirmed the inherent rights of First Nations, Inuit and Mtis governments to assert jurisdiction over child welfare for Indigenous children.^4^ Indigenous governing bodies have begun to create laws, deliver services and redesign child welfare systems so that they are self-determined and rooted in culturally specific approaches to care.^3^,^4^

In Canada, a small proportion of children involved in the child welfare system are in out-of-home care.^5^ This includes children placed with extended family, in foster homes or in group or institutional settings.^5^-^7^ Many high-income countries collect administrative data that are used to report on indicators about child welfare services, including the number of children in out-of-home care.^8^-^12^ Analysis of such data at the national level in Canada has been limited,^13^,^14^ although several studies have estimated the size of the population of children in out-of-home care.

According to the *Health Behaviour in School-Aged Children (HBSC) Survey*, which covers a nationally representative sample of children aged 11, 13, and 15 years, 2.4% of children in Canada were living in a foster home or a “children’s home” or were cared for by a non-parental family member in 2017/2018.^15^ In 2019, the *First Nations/Canadian Incidence Study of Reported Child Abuse and Neglect* (FN/CIS) found that 15071 children (First Nations and non-Indigenous) were placed in out-of-home care following a new child protection investigation.^5^ Estimates calculated based on data from the 2021 national census indicate that there were 26680 children aged 0 to 14 years in foster care (4.45 per 1000 children).^16^ Previous analyses of administrative data estimated that the number of children in out-of-home care peaked at 64755 in 2009 (8.8 per 1000)^17^ and then declined to 59283 (8.2 per 1000) in 2019.^7^ While each of these sources provides a count of a subpopulation of children in out-of-home care, they fall short of being comprehensive national estimates because of their objective, study design, definitions or coverage.^7^,^16^,^18^

Evidence shows that children in out-of-home care face greater risks for poor health, social and educational outcomes because of adverse early life experiences such as maltreatment and poverty.^19^ Placing children in family-based care environments can reduce risks of mental health problems and other negative consequences associated with maltreatment.^20^ However, the experience of being in out-of-home care itself can have independent deleterious effects over the life course,^19^,^21^,^22^ and children in group or institutional settings in particular experience elevated developmental, cognitive and social risks.^11^,^23^,^24^ The tension between these realities is especially difficult to negotiate in child welfare policy and practice in Canada because First Nations, Inuit, Mtis, Black and other communities made vulnerable by structural inequities are disproportionately harmed by involvement in the child welfare system.^25^-^28^

As a part of the public health approach to child welfare,^18^,^29^,^30^ population-based data about children in out-of-home care are necessary to assess trends over time, understand drivers of social and health inequities, and examine outcomes for children and families. Such data can inform policy decisions, interventions and community action.^13^,^29^,^31^,^32^

To expand on previous studies^5^,^7^,^16^,^17^,^33^,^34^ and strengthen the epidemiological evidence on children in out-of-home care,^29^ we analyzed national administrative child welfare data in Canada. The objectives were to: (1) describe the population of children* in out-of-home care; (2) estimate the rate of out-of-home care overall and by province/territory, year, sex/gender, age group, and placement type; and (3) compare rates by province/territory, sex/gender, age group, and placement type. 

*In the objectives and elsewhere, we refer to data on “children” for brevity, but this population also includes youth, unless otherwise specified.

## Methods

We conducted a cross-sectional analysis of data from the Canadian Child Welfare Information System (CCWIS). The CCWIS is a national administrative database derived from demographic, clinical and legal information that is routinely collected and recorded in electronic case management systems by frontline staff as a part of delivering child welfare services. Following several years of partnership building as well as a feasibility assessment,^29^,^35^ the CCWIS was developed by the Public Health Agency of Canada (PHAC) to address national child welfare data gaps and monitor population-level indicators across person, place and time. CCWIS data can support policy and program decisions related to child and family well-being, and may be used for evaluating the impact of legislative, policy, and social changes on the child welfare system.

***Data source***

The CCWIS contains count (also called “aggregate”) and record-level data about children in out-of-home care. Data were obtained from all 13 provincial and territorial departments responsible for child welfare services and were derived from one of three sources: (1) publicly available aggregate data from annual reports and data dashboards (“public data”); (2)custom tabulated aggregate data (“custom data”); and (3) de-identified record-level data (“record-level data”). 

Several approaches were used to assemble CCWIS data. PHAC epidemiologists created a standardized data collection form to extract counts and information about definitions and parameters from online reports or dashboards recommended by each provincial and territorial child welfare department. Data obtained from public sources were shared with quality assurance and data management staff in each jurisdiction for review, correction and validation.

Because stratified data were not publicly available from most provincial and territorial child welfare departments, PHAC requested custom tabulations by year, sex/gender, age group and placement type using an adapted version of the standardized data collection form. The adapted form included CCWIS definitions and eligibility criteria, along with predefined categories based on previous studies,^5^,^36^ and prompts to describe the corresponding parameters. During the process of validating the public data, all provinces and territories were invited to submit custom data as an enhanced alternative, to be shared with PHAC on a voluntary basis.

Record-level data from the Northwest Territories were obtained through a data sharing agreement between the Government of Northwest Territories (GNWT) and PHAC. The agreement was developed for both a regional data initiative (the Pan Territorial Data Project)^29^ and the CCWIS; this agreement permitted the transfer of de-identified data to PHAC and the use of data for statistical purposes. Since the public data about children in out-of-home care for the territory were based on the total for the fiscal year, PHAC and the GNWT aggregated the record-level data to generate stratified point-in-time counts. This step helped harmonize the data for the national analysis and improve the comparability of the territory’s rates with other jurisdictions.

Overall, the CCWIS contains public data from six provinces and territories and Indigenous Services Canada (ISC), custom data from five provinces, a mix of public and custom data from one province, and record-level data from one territory; coverage for each jurisdiction varies by year, demographics and placement type (Table1). Data from Indigenous child welfare agencies were included in the CCWIS only if these data were routinely collected and reported by a provincial or territorial jurisdiction or by ISC. Data from all jurisdictions include First Nations, Inuit and Mtis children. However, we did not calculate Indigenous-specific rates of out-of-home care for the present analysis because we did not have permission from Indigenous or provincial/territorial partners to do so, nor did we have access to distinction-based data for most jurisdictions. All CCWIS data are considered “secondary data” because the source information was originally generated for the purpose of delivering services, not for population statistics. The CCWIS is updated when additional data from participating jurisdictions are shared with PHAC. 

**Table 1:** CCWIS data coverage by province and territory, Canada, 2013/2014–2021/2022

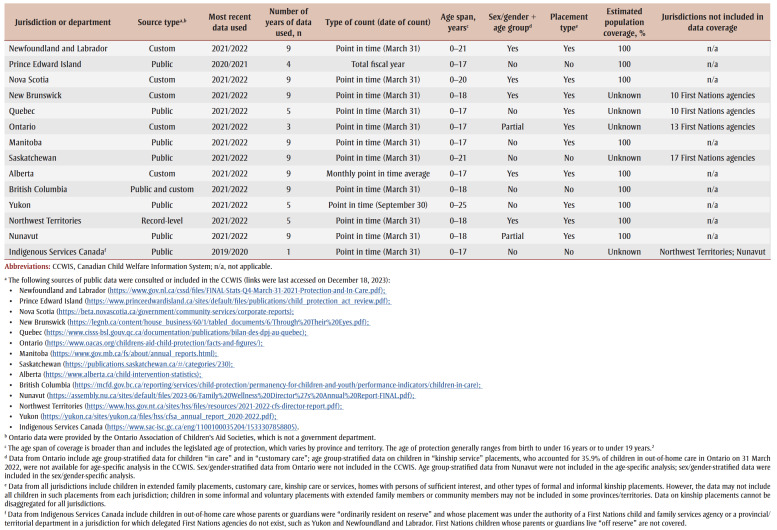

For this analysis, we extracted CCWIS data for the fiscal year period, 2013/2014 to 2021/2022 (1 April 2013 to 31 March 2022, inclusive), as available. Most data used in this analysis (99.64%) were derived from public or custom data. The results of this analysis may differ from the information that is publicly reported by provinces/territories (see Table1) due to differences between the national definition of out-of-home care and the definitions used in each jurisdiction.

***Definition of out-of-home care***

In the CCWIS, children in out-of-home care are those placed in a setting other than their usual home for any reason and for any length of time. Owing to differences in legislation, funding and policy, the specific parameters for placement eligibility vary by province and territory.^2^,^3^,^37^ In alignment with global approaches to statistics on children in “alternative care,”^6^,^10^,^12^ the CCWIS has a broad definition of out-of-home care in order to cover children in both formal and informal placements, with any legal status, and in family-based care, group care or other placement settings.

For CCWIS data, the age span of coverage includes but is broader than the legislated age of protection, which is from birth to under 16 years or up to under 19 years.^2^ Some youth receive placement services under voluntary agreements that can extend to 25 years of age.^2^ We adjusted for differences in age span by matching the age parameters of the population data (denominator) with the jurisdiction-specific coverage age span (Table1) for the count data (numerator), and by restricting overall and stratified analyses to count data that were reported by at least four provinces and territories.

As in other child welfare data systems,^6^,^8^,^10^,^38^,^39^ CCWIS data are based on a point-in-time count. For each fiscal year, children in out-of-home care were enumerated only if they were in a placement on 31 March.^7^ Three jurisdictions—Prince Edward Island, Alberta and Yukon—did not report a count on 31 March; alternative counts were treated as a proxy for March 31 counts.

***Variables***

We analyzed data about children in out-of-home care across five variables: province/territory, year, sex/gender, age group and placement type. The variable “province/territory” indicates the jurisdiction that provided the data to the CCWIS. For most children, this was the province/territory where they were placed. The variable “year” refers to a fiscal year, from 1 April to 31 March.

The CCWIS does not distinguish between sex assigned at birth and gender identity because these distinctions were not evident in the data provided by provinces and territories. For the present analysis, we referred to “sex/gender” and stratified by female and male. For the variable “age group,” we used the categories less than 1year (0–11 months; infants), 1 to 3 years, 4 to 7 years, 8 to 11 years, 12 to 15 years, 16 to 17 years and 18 to 21 years (to 25years in Yukon). Child age was as of the date of enumeration (31 March in most jurisdictions; see Table1. 

Based on previous analyses from Canada^5^ and abroad,^10^-^12^,^38^ we used four placement type categories: kinship home, foster home, group care and other. We refer to kinship and foster homes together as family-based care. These categories differ from the naming conventions used in some provinces and territories and communities; nomenclature for settings where children in out-of-home care reside is changing as service providers develop an increasingly broad range of placement options. Our terminology reflects the primary categories currently applied in most jurisdictions (see Table 2).

**Table 2:** Placement type definitions in the CCWIS

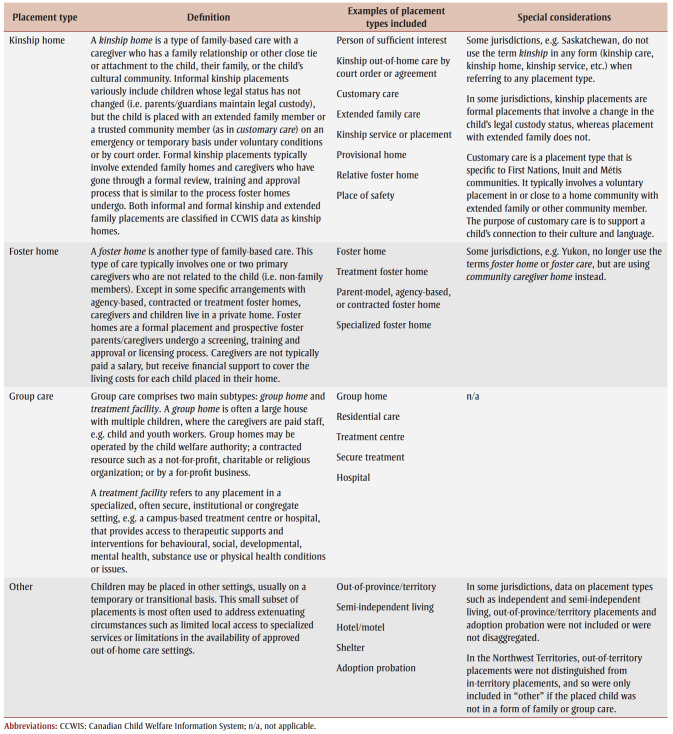

***Statistical analysis***

We used frequencies and percentage to describe the population of children in out-of-home care. We calculated rates overall and by province/territory, year, sex/gender, age group and placement type. With more detailed data from selected provinces and territories, we were able to conduct stratified analyses.

Rates were estimated by dividing the number of children in out-of-home care on 31 March by the total number of children in a population. Population data were obtained from Statistics Canada’s annual intercensal estimates^40^ and included jurisdiction-specific parameters for age to account for variations in age span of coverage in each province and territory (Table1). All rates were reported with 95% confidence intervals (CIs), calculated using the exact method.^41^

For a sensitivity analysis, we combined data from the provinces and territories with data from ISC. We could not identify or exclude children who may have been counted in both provincial/territorial and ISC data. However, pooling sources allowed us to include additional data about children served by First Nations agencies in four provinces (Table1) who were not otherwise covered and estimate a maximum national rate of out-of-home care. Public count data from ISC were available at the national level only.

For comparisons, we calculated rate ratios (RR) with 95% CIs and used the national rate with and without ISC data as the reference group. The analysis was conducted using SAS Enterprise Guide version 7.1 (SAS Institute, Cary, NC, USA).

***Ethics***

This analysis was approved by PHAC’s Science Review Committee and underwent a Health Canada/PHAC privacy impact assessment. Legislative authority for the development and analysis of CCWIS data is provided by section 4 of the *Department of Health Act*^42^ and section 3 of the *Public Health Agency of Canada Act.*^43^ Our analysis was exempt from research ethics board approval as per Canada’s *Tri-Council Policy Statement: Ethical Conduct for Research Involving Humans* because we used the data for public health surveillance.^44^

In recognition of the guidelines for research and the standards for data governance in Indigenous communities,^44^-^48^ we took steps to understand the priorities of Indigenous organizations in order to develop CCWIS data and conduct the analysis. This involved inviting representatives from National Indigenous Organizations to join the PHAC Working Group that oversees the CCWIS (see the Acknowledgements section); liaising with established groups or networks involved in Indigenous child welfare data governance; hosting engagement sessions with Indigenous organizations to understand how CCWIS might address the need for distinction-based data and be governed through multilateral partnerships; and sharing updates and seeking feedback on CCWIS activities through presentations, meetings and the review of preliminary results and draft materials. Efforts to build partnerships with First Nations, Inuit and Mtis organizations are ongoing.

## Results

An estimated 61104 children were in out-of-home care in Canada in fiscal year 2021/2022 (Table 3). The national rate of out-of-home care was 8.24 per 1000. When ISC data were included in the calculation, the estimated count was 70434 with a rate of 9.50 per 1000. The rate difference between the estimates was 1.26 per 1000 (95% CI: 1.16–1.36) and the percentage difference was 14.2%.

**Table 3:** Number, percentage, rate and rate ratio of children in out-of-home care, by province/territory, sex/gender, age group, and placement type, Canada, 2021/2022

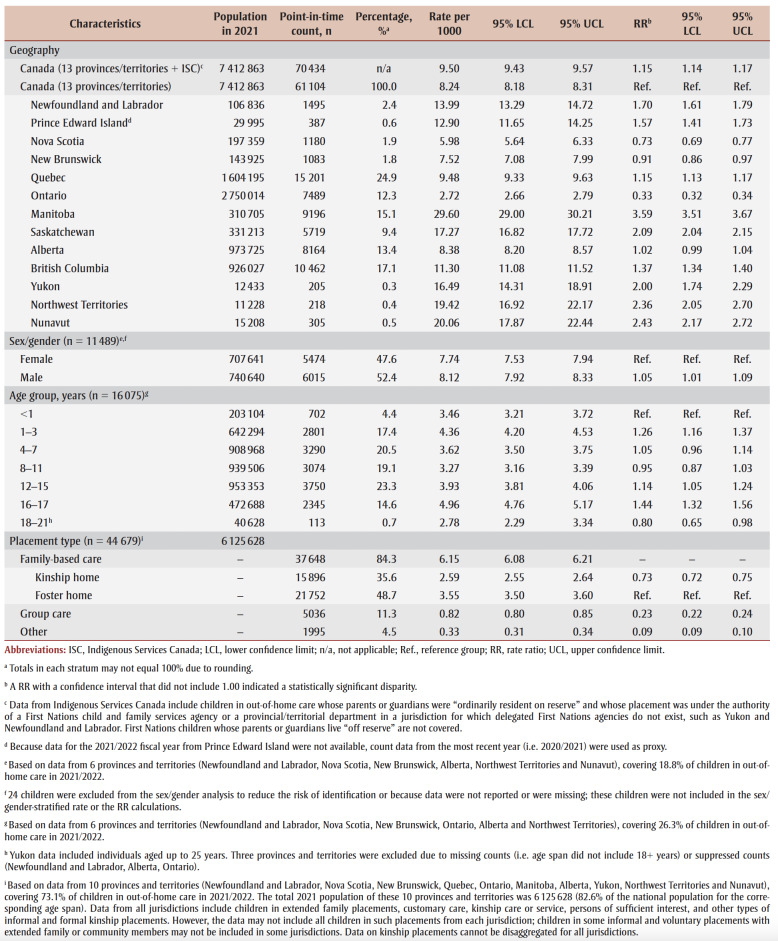

**Figure 1:**
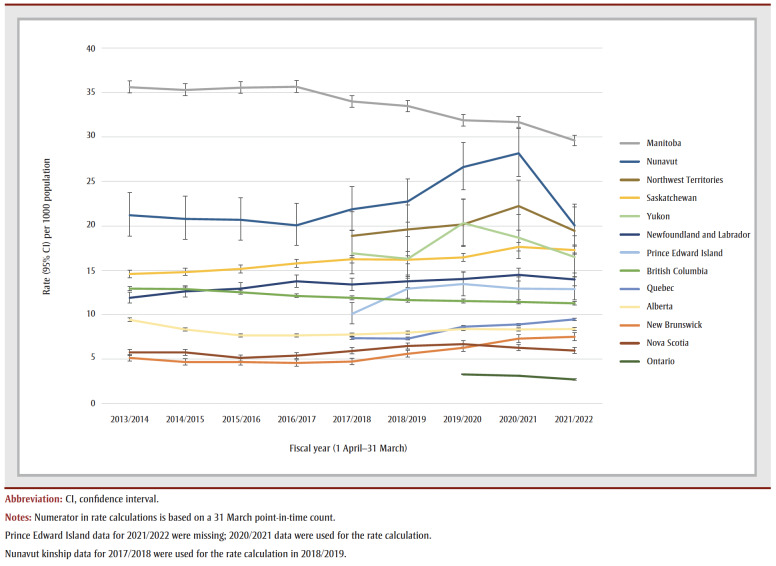
Rates of children in out-of-home care, by province/territory and fiscal year, Canada, 2013/2014–2021/2022

**Figure 2:**
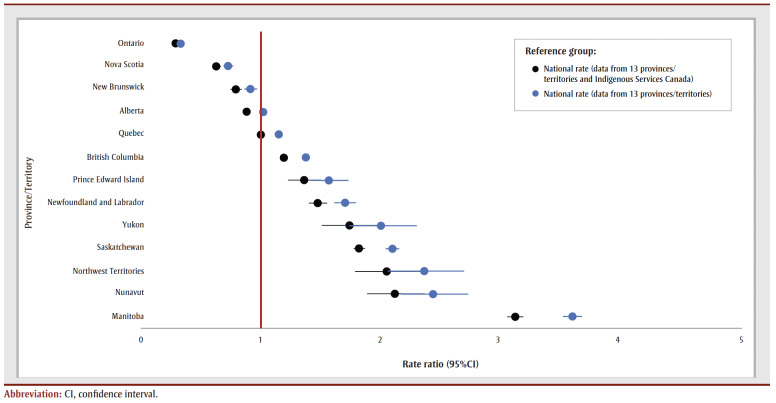
Rate ratios of children in out-of-home care, by province/territory, Canada, 2021/2022

Rates of out-of-home care in 2021/2022 varied by province and territory (Table 3, Figure 1 and Figure 2). Rates were lowest in Ontario (2.72 per 1000) and Nova Scotia (5.98 per 1000) and highest in Manitoba (29.60 per 1000) and Nunavut (20.06 per 1000). During the fiscal year period 2013/2014 to 2021/2022, rates declined in Manitoba and British Columbia, increased in Newfoundland and Labrador, New Brunswick, Quebec and Saskatchewan, and remained relatively stable in the other provinces and territories (Figure 1). 

Provincial/territorial rates were 2 to 3times higher than the national rate in Yukon, Saskatchewan, Northwest Territories, Nunavut and Manitoba and lower in New Brunswick, Nova Scotia and Ontario ( Table 3; Figure 2). The size of the disparities varied depending on which national rate estimate was used.

Based on data from the six provinces and territories with data on sex/gender, males accounted for 52.4% of children in out-of-home care in 2021/2022 (Table 3). The out-of-home care rate for males was also slightly higher than the rate for females (RR = 1.05; 95% CI: 1.01–1.09).

Of the six provinces and territories with age group–specific data, children aged 12 to 15 years accounted for the largest percentage (23.3%) of children in out-of-home care (Table 3). Of all children in out-of-home care in 2021/2022, 84.7% were younger than 16 years. Rates were highest for children aged 1 to 3 years and 16 to 17 years, and slightly but significantly higher (RR = 1.44 and 1.26, respectively) than the rate for infants.

**Figure 3:**
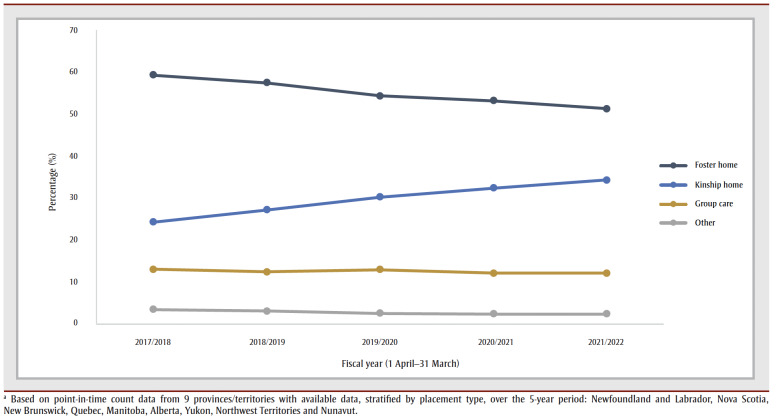
Percentages of children in out-of-home care, by placement type in Canada, fiscal year 2017/2018 to 2021/2022

In the ten provinces and territories with data on placement type for 2021/2022, family-based care accounted for 84.3% of children in out-of-home care; the majority of these placements were foster homes. Group care accounted for 11.3% of placements (Table 3). Based on data from nine provinces and territories, during the 5-fiscal year period from 2017/2018 to 2021/2022, the overall percentage of children in foster homes decreased and the percentage in kinship placements increased (Figure 3).

## Discussion

We used national administrative child welfare data to examine rates of out-of-home care for children in Canada. An estimated 61104 children were in out-of-home care in 2021/2022 (not including ISC data). Rates were significantly higher than the national rate in nine provinces and territories, and significantly lower in three; this changed slightly when the national rate including ISC data was used as the reference Figure 2). The low (8.24 per 1000) and high (9.50 per 1000) rate estimates from the CCWIS were similar to the rate of out-of-home care in Australia (8.1 per 1000 in 2020),^8^ higher than the rate in the United States (5.8 per 1000 in 2019)^39^ and within the range for countries in Europe and Central Asia (1–21 per 1000 in 2021).^10^,^38^

The 2021/2022 rate of out-of-home care that did not include ISC data (8.24 per 1000) was comparable to previous estimates from 2009/2010 (8.8 per 1000)^17^ and 2019/2020 (8.2 per 1000)^7^ derived from similar data sources. However, our estimate with ISC data (9.50 per 1000) suggests that the rate may have increased or had been previously underestimated. These findings contrast with the decline in the rate of children in foster care shown by census data: from 4.93 per 1000 in 2016 to 4.45 in 2021.^16^ FN/CIS data showed a somewhat different pattern, with an increase in placement rates between 1998 and 2008 (2.67 to 3.26 per 1000)^36^ and a decline from 2008 to 2019 (to 2.59 per 1000). At the provincial/territorial level, rates over time varied by jurisdiction. The factors behind this heterogeneity across data sources and across geographies are not evident, but warrant further analysis.

The CCWIS rate for family-based care in 2021/2022 (6.15 per 1000) was somewhat similar to the 2021 Census estimate for children in foster homes (4.45 per 1000).^16^ The difference may be because the census rate did not include some children in kinship homes or customary care or placed informally with extended family members.^16^ The FN/CIS found that 48% of children placed in out-of-home care after a child protection investigation were in an informal kinship home or customary care; 44% were in foster homes (14% kinship, 30% non-relative).^5^ These findings differed from CCWIS results. Nonetheless, 92% of children in out-of-home care were in some type of family-based setting in the FN/CIS.^5^ This is broadly consistent with the 84.3% found in our analysis,^5^ and similar to findings from a 2023 Ontario study.^23^

The discrepancy between the FN/CIS and the CCWIS likely reflects different percentages of children in group care—6% in the FN/CIS^5^ versus 12% in our analysis—and missing data on informal kinship placements for some jurisdictions in the CCWIS. Because the FN/CIS captured data early in the child welfare investigation process, it may have been more likely than administrative data to identify children in informal kinship placements. Owing to the use of mostly aggregate data and the limited ability to disaggregate public sources in the CCWIS, it is also possible that some formal kinship placements were misclassified as foster homes, thereby inflating the prevalence of this placement type.

***National child welfare data and Indigenous data governance***

Child welfare systems in Canada have an important role in upholding children’s rights to safety and security and protecting them from maltreatment.^1^,^3^ However, these are colonial systems with abiding legacies of institutional abuse and discrimination against Indigenous, Black and other racialized communities^25^-^28^,^49^,^50^ who continue to be overrepresented among children in out-of-home care.^5^,^27^ With these realities in mind, we recognize that CCWIS data are neither neutral nor objective. The information that formed the basis of the data used in our analysis was generated by interventions that can cause harm by separating children from their families and communities and disconnecting them from their culture. The disproportionality of this harm is one of the ways that CCWIS data are imbued with the racism that is manifest in child welfare.^50^

One of the risks in epidemiology with secondary data is that methods and results become detached from the social history and experiences of the people and communities that are represented by the data. We attempted to mitigate this risk during the development and analysis of CCWIS data by being transparent about the information we were using, sharing updates on our decisions and progress, and inviting input and participation from Indigenous organizations, provincial/territorial ministries and federal departments. This outreach helped align our analytical objectives with the priorities of child welfare and Indigenous partners, develop and test a governance model for national administrative data, and contextualize the findings. These efforts are important because the analysis of CCWIS data is meant to be an ongoing activity that serves as a resource in child welfare and public health decision- making.

With a long-term approach to social and institutional licensing, we also sought to minimize the ways our methodology may have contravened guidelines for the use of data related to Indigenous Peoples and balance this with the value the information can provide. Drawing on instructive examples from research,^5^,^51^ we will continue to collaborate with provincial/territorial partners, First Nations, Inuit and Mtis organizations, and rights-holders and communities to find ways to respect and operationalize the principles of Indigenous data sovereignty^47^ in the CCWIS.

***Strengths and limitations***

Our analysis has several strengths. The geographic and population coverage of CCWIS data were high: we had data from all provinces and territories; the inclusion of custom and record-level data enhanced consistency in coverage and definitions; data from nine jurisdictions had full population coverage; and the placement types we employed were broadly comparable (Table 2), with placement type-stratified data from 10 provinces and territories covering 73.1% of the national population of children in out-of-home care. By using ISC data for the sensitivity analysis, we had near-complete capture of jurisdictions that collect data on children in out-of-home care.

A limitation of our analysis is that jurisdictions’ definitions of out-of-home care vary by child age, legal status and authority, types of placements, relationship to caregivers, duration, and cultural and geographic context. Because the CCWIS data we used were based primarily on aggregate data, there were relatively few opportunities for harmonizing definitions. We attempted to lessen the effects of definitional differences by noting variations in coverage (Table 1) and ensuring the population data (denominator) in rate calculations matched the parameters of the number of children in out-of-home care (numerator). Definitional issues were also partially offset by using a standardized data collection form. 

For the sensitivity analysis, to estimate a maximum national rate, we included ISC data that covered First Nations child welfare agencies. This may have helped to account for variations in undercoverage for specific provinces, such as Ontario, but the impact on provincial/territorial rates is unclear. Ongoing collaborations with partners provide an opportunity to further refine definitions and data standards, and expand data coverage.

Another limitation was related to the use of aggregate data, which restricted our ability to carry out in-depth data quality assessments and conduct stratified analyses along dimensions of equity. Record-level administrative data from more provinces and territories would enable the identification of individual risks in child welfare,^52^,^53^ research on the pathways to out-of-home care and beyond^21^,^54^ and an assessment of the extent of missing data and double-counting.

CCWIS data have gaps in coverage for specific populations (such as First Nations children on reserve or under the jurisdiction of First Nations agencies), some years (especially before 2013) and demographic and service variables (such as sex/gender, age and placement type). For example, data on children in informal or emergency placements with extended family may be missing from the CCWIS data we analyzed in some jurisdictions. This and other coverage issues likely contributed to the national rate and the rates for selected provinces and territories being underestimated. A related challenge was that sex/gender and age-specific estimates were based on data from six provinces and territories, representing only 18.8% and 26.3% of all children in out-of-home care in Canada, respectively. Therefore, these results may not reflect national patterns and should be interpreted with caution. Including data disaggregated by sex/gender, age, Indigenous identity, race/ethnicity, geography and placement type from all provinces and territories in the CCWIS will help clarify epidemiological patterns and identify differential risks among specific subgroups.

Finally, our estimates pertained to a single point in time in each year (31 March). This is a common method of reporting the number of children in out-of-home care,^8^-^10^,^13^ but it underestimates the annual total. Some children move in and out of care, often for short durations,^23^ and may not be counted on a specific date. An alternative is a “period” count that refers to the number of children in out-of-home care for at least one night any time during the year. Point-in-time and period counts may be correlated, but the proportionate difference between them is not clear and warrants examination. Expanding CCWIS coverage to include out-of-home care admissions and discharges, duration, number of moves, legal status and reason for placement, along with data on child welfare referrals, investigations, services and youth supports, would improve the breadth and depth of indicators that can be generated.

***Implications for public health monitoring and policy***

In 2015, the Truth and Reconciliation Commission called on the federal, provincial and territorial governments to “publish annual reports on the number of Aboriginal children (First Nations, Inuit and Mtis) who are in care compared with non-Aboriginal children […].”^25^^,p.140^ The need for this information was further underscored by the Calls for Justice from the National Inquiry into Missing and Murdered Indigenous Women and Girls.^26^ With Indigenous partners and distinctions-based data, CCWIS data could be used to directly address the Truth and Reconciliation Commission’s second Call to Action^25^ and track progress of the federal child welfare legislation’s objective of reducing the number of Indigenous children in care.^4^

The development of national data on children in out-of-home care is a first step in improving the transparency and accessibility of child welfare data. With a co-developed governance structure, data sharing agreements and expanded coverage, the CCWIS will be able to create additional national indicators about the child welfare system, harmonize definitions across jurisdictions, improve data quality and disaggregation, and generate population-based evidence on children’s health and well-being. By strengthening CCWIS data, governments, agencies, researchers and communities can better monitor inequalities, track the health and social outcomes of children and families, and evaluate and inform policies and interventions.

## Conclusion

We used national child welfare data to examine rates of out-of-home care among children in Canada. More than 61000 children were in out-of-home care in 2021/2022; rates varied substantially by province and territory, and family-based care was the most common type of placement. Our analysis demonstrated that a working definition of out-of-home care can be applied to multiple sources of administrative data to measure broadly similar types of placements, and that these data can, in turn, be used to generate national indicators about children and families involved in the child welfare system.

## Acknowledgements

Thank you to the following individuals and organizations for the support and guidance in conducting this study: Mary Sue Devereaux, Joanna Odrowaz and Anna Olivier for editing; Dawn-Li Blair, Natalie Gabora, Prateek Sharma, Margot Shields and colleagues in the Surveillance Systems and Data Management Division, Centre for Surveillance and Applied Research, PHAC; Shari Fitzgerald, Mark Griffin, Deanne O’Brien and Shuhana Shahnaz, Child and Youth Services Branch, Department of Children, Seniors and Social Development, Government of Newfoundland and Labrador; staff from the Department of Social Development and Seniors, Government of Prince Edward Island; staff from the Department of Community Services, Government of Nova Scotia; Lorraine Hill, Association of Native Child and Family Services Agencies of Ontario; staff from the Ministry of Children, Community and Social Services, Government of Ontario; Michelle Gingrich, Jon Van Tuyl, and Gloria Miguel, Ontario Association of Children’s Aid Societies; Fabien Pernet, Makivvik; Greg Maclean, Angela Miller and Credell Simeon, Ministry of Social Services, Government of Saskatchewan; France Cormier and Steven Yong, Ministry of Children and Family Development, Government of British Columbia; staff from the Family and Children’s Services Branch, Department of Health and Social Services, Government of Yukon; Cheuk Pang and Amanda White, Child and Family Services, Department of Health and Social Services, Government of Northwest Territories; Molly Rasmussen, First Nations Child & Family Caring Society; Natasha Steinback, Mtis National Council; Danick Valle Blanchard and staff from the Data Accountability and Performance Unit, Child and Family Services, Indigenous Services Canada; Melody Morton Ninomiya, Wilfrid Laurier University; members of the Child Maltreatment Surveillance and Research Working Group (Tracie Afifi, University of Manitoba; Pierre-Jean Alasset, Indigenous Services Canada; Erin Aylward, PHAC; Marni Brownell, University of Manitoba; Peter Dudding, Loyalist College; Andrea Gonzalez, McMaster University; Shannon Hurley, PHAC; Harriet MacMillan [Chair], McMaster University; Susan McDonald, Justice Canada; Katherine Minich, Inuit Tapiriit Kanatami and Carleton University; Kenn Richard, Kenn Richard/Associates; Vicki Scott, Canadian Institute of Health Information; Marcel St. Onge, Mtis National Council; Cassandra Yantha, Inuit Tapiriit Kanatami); the Provincial and Territorial Directors of Child Welfare (Joanne Cotter, Child and Youth Services Branch, Department of Children, Seniors and Social Development, Government of Newfoundland and Labrador; Directors from the Department of Community Services, Government of Nova Scotia; Lorna Hanson, Child and Youth Services Division, Department of Families, Government of Manitoba; Janice Colquhoun, Ministry of Social Services, Government of Saskatchewan; Elden Block, Ministry of Children and Family Services, Government of Alberta; James Wale, Ministry of Children and Family Development, Government of British Columbia; Colette Prevost, Child and Family Services, Department of Health and Social Services, Government of Northwest Territories [Colette also serves as the representative for the Directors of Child Welfare on the Child Maltreatment Surveillance and Research Working Group]; and representatives from the Department of Family Services, Government of Nunavut). Thank you as well to the anonymous peer reviewers and to the editorial and production teams at the journal.

## Funding

The Public Health Agency of Canada provided funding for the analysis.

## Conflicts of interest

The authors have no conflicts of interest to declare.

## Authors’ contributions and statement

NJP: Conceptualization, methodology, investigation, data curation, writing – original draft, visualization, supervision, project administration.

AMO: Conceptualization, methodology, software, validation, formal analysis, investigation, data curation, writing - review & editing, visualization.

NT: Conceptualization, methodology, writing – original draft, supervision.

WH: Methodology, writing – original draft, supervision, project administration.

AM: Conceptualization, methodology, validation, investigation, resources, data curation, writing – review & editing.

LC: Methodology, software, validation, formal analysis, data curation, writing – review & editing, visualization.

AC: Methodology, writing – original draft.

MT: Software, validation, writing – review & editing.

CZ: Investigation, data curation, writing – review & editing.

CL: Conceptualization, writing – review & editing, supervision.

LT: Conceptualization, methodology, resources, writing – original draft, visualization, supervision, project administration, funding acquisition.

The content and views expressed in this article are those of the authors and do not necessarily reflect those of the Government of Canada or of the Government of Alberta.
